# Insomnia associated with neutrophil/lymphocyte ratio in female patients with schizophrenia

**DOI:** 10.1192/j.eurpsy.2021.2131

**Published:** 2021-08-13

**Authors:** G. Paniagua, L. González-Blanco, F. Dal Santo, C. Martínez-Cao, C. Moya-Lacasa, M. Valtueña-García, E. Martín Gil, L. García-Alvarez, P.A. Saiz, M.P. García-Portilla, J. Bobes

**Affiliations:** 1 Department Of Psychiatry, University of Oviedo, Oviedo, Spain; 2 Psychiatry, SESPA Mental Health Services of Principado de Asturias, OVIEDO, Spain; 3 Csm Eria, Servicio Salud Principado Asturias, University of Oviedo, Asturias, Oviedo, Asturias, Spain; 4 Neuroscience And Sense Organs, ISPA HEALTH RESEARCH INSTITUTE OF THE PRINCIPALITY OF ASTURIAS, Oviedo, Spain; 5 Deparment Of Psychiatry, University of Oviedo/CIBERSAM, Oviedo, Spain; 6 Psychiatry, Hospital Universitario Central de Asturias, Oviedo, Spain

**Keywords:** female, schizophrénia, Insomnia, Inflammation

## Abstract

**Introduction:**

Worse sleep quality and increased inflammatory markers in women with schizophrenia (Sch) have been reported (Lee et al. 2019). However, the physiological mechanisms underlying the interplay between sleep and the inflammatory pathways are not yet well understood (Fang et al. 2016).

**Objectives:**

Analyze the relationship between Neutrophil/Lymphocyte (NLR), Monocyte/Lymphocyte (MLR) and Platelet/Lymphocyte (PLR) ratios, and insomnia in Sch stratified by sex.

**Methods:**

Final sample included 176 Sch patients (ICD-10 criteria) [mean age: 38.9±13.39; males: 111(63.1%)]. Assessment: PANSS, Calgary Depression Scale (CDSS), and Oviedo Sleep Questionnaire (OSQ) to identify a comorbid diagnosis of insomnia based on ICD-10. Fasting counting blood cell were performed to calculate ratios. Statistics: U Mann-Whitney, logistic regression.

**Results:**

Insomnia as comorbid diagnosis was present in 22 Sch (12.5%) with no differences between sex [14 males (12.6%), 8 females (12.3%)], neither in their age. Female patients with insomnia showed increased NLR [2.44±0.69 vs. 1.88±0.80, U=122.00 (p=0.034)]. However, no differences in PLR and MLR were found, neither in any ratio in males. Regression models using insomnia as dependent variable and covariates (age, PANSS-positive, PANSS-negative, CDSS) were estimated. Females: presence of insomnia was associated with NLR [OR=3.564 (p=0.032)], PANSS-positive [OR=1.263 (p=0.013)] and CDSS [OR=1.198 (p=0.092)]. Males: only PANSS-positive [OR=1.123 (p=0.027)] and CDSS scores [OR=1.220 (p=0.005)] were associated with insomnia.

**Conclusions:**

NLR represent an inflammatory marker of insomnia in Sch but only in female patients. Improving sleep quality in these patients could help to decrease their inflammatory response.

**Disclosure:**

No significant relationships.

